# Role of Phagocytosis in the Pro-Inflammatory Response in LDL-Induced Foam Cell Formation; a Transcriptome Analysis

**DOI:** 10.3390/ijms21030817

**Published:** 2020-01-27

**Authors:** Alexander N. Orekhov, Nikita G. Nikiforov, Vasily N. Sukhorukov, Marina V. Kubekina, Igor A. Sobenin, Wei-Kai Wu, Kathy K. Foxx, Sergey Pintus, Philip Stegmaier, Daria Stelmashenko, Alexander Kel, Alexei N. Gratchev, Alexandra A. Melnichenko, Reinhard Wetzker, Volha I. Summerhill, Ichiro Manabe, Yumiko Oishi

**Affiliations:** 1 Laboratory of Angiopathology, Institute of General Pathology and Pathophysiology, 8 Baltiiskaya Street, 125315 Moscow, Russia; 2 Laboratory of Infection Pathology and Molecular Microecology, Institute of Human Morphology, 3 Tsyurupa Street, 117418 Moscow, Russia; 3 Laboratory of Medical Genetics, Institute of Experimental Cardiology, National Medical Research Center of Cardiology, 15A 3-rd Cherepkovskaya Street, 121552 Moscow, Russia; 4 Centre of Collective Usage, Institute of Gene Biology, Russian Academy of Sciences, 34/5 Vavilova Street, 119334 Moscow, Russia; 5 Department of Internal Medicine, National Taiwan University Hospital, Bei-Hu Branch, Taipei 10002, Taiwan; 6 Kalen Biomedical, LLC, Montgomery Village, MD 20886, USA; 7 BIOSOFT.RU, LLC, 630090 Novosibirsk, Russia; 8 Institute of Computational Technologies, 630090 Novosibirsk, Russia; 9 geneXplain GmbH, 38302 Wolfenbüttel, Germany; 10 Institute of Chemical Biology and Fundamental Medicine, 630090 Novosibirsk, Russia; 11 N. N. Blokhin National Medical Research Center of Oncology, 24 Kashirskoye sh., 115478 Moscow, Russia; 12 Department of Anaesthesiology and Intensive Care Medicine, University Hospital Jena, Am Klinikum 1, D-07747 Jena, Germany; 13 Department of Basic Research, Institute for Atherosclerosis Research, 121609 Moscow, Russia; 14 Department of Aging Research, Graduate School of Medicine, Chiba University, Chiba 263-8522, Japan; 15 Department of Biochemistry & Molecular Biology, Nippon Medical School, Tokyo 113-8602, Japan

**Keywords:** atherosclerosis, transcriptome, lipoprotein, phagocytosis, innate immunity

## Abstract

Excessive accumulation of lipid inclusions in the arterial wall cells (foam cell formation) caused by modified low-density lipoprotein (LDL) is the earliest and most noticeable manifestation of atherosclerosis. The mechanisms of foam cell formation are not fully understood and can involve altered lipid uptake, impaired lipid metabolism, or both. Recently, we have identified the top 10 master regulators that were involved in the accumulation of cholesterol in cultured macrophages induced by the incubation with modified LDL. It was found that most of the identified master regulators were related to the regulation of the inflammatory immune response, but not to lipid metabolism. A possible explanation for this unexpected result is a stimulation of the phagocytic activity of macrophages by modified LDL particle associates that have a relatively large size. In the current study, we investigated gene regulation in macrophages using transcriptome analysis to test the hypothesis that the primary event occurring upon the interaction of modified LDL and macrophages is the stimulation of phagocytosis, which subsequently triggers the pro-inflammatory immune response. We identified genes that were up- or downregulated following the exposure of cultured cells to modified LDL or latex beads (inert phagocytosis stimulators). Most of the identified master regulators were involved in the innate immune response, and some of them were encoding major pro-inflammatory proteins. The obtained results indicated that pro-inflammatory response to phagocytosis stimulation precedes the accumulation of intracellular lipids and possibly contributes to the formation of foam cells. In this way, the currently recognized hypothesis that the accumulation of lipids triggers the pro-inflammatory response was not confirmed. Comparative analysis of master regulators revealed similarities in the genetic regulation of the interaction of macrophages with naturally occurring LDL and desialylated LDL. Oxidized and desialylated LDL affected a different spectrum of genes than naturally occurring LDL. These observations suggest that desialylation is the most important modification of LDL occurring in vivo. Thus, modified LDL caused the gene regulation characteristic of the stimulation of phagocytosis. Additionally, the knock-down effect of five master regulators, such as *IL15, EIF2AK3, F2RL1, TSPYL2*, and *ANXA1*, on intracellular lipid accumulation was tested. We knocked down these genes in primary macrophages derived from human monocytes. The addition of atherogenic naturally occurring LDL caused a significant accumulation of cholesterol in the control cells. The knock-down of the *EIF2AK3* and *IL15* genes completely prevented cholesterol accumulation in cultured macrophages. The knock-down of the *ANXA1* gene caused a further decrease in cholesterol content in cultured macrophages. At the same time, knock-down of *F2RL1* and *TSPYL2* did not cause an effect. The results obtained allowed us to explain in which way the inflammatory response and the accumulation of cholesterol are related confirming our hypothesis of atherogenesis development based on the following viewpoints: LDL particles undergo atherogenic modifications that, in turn, accompanied by the formation of self-associates; large LDL associates stimulate phagocytosis; as a result of phagocytosis stimulation, pro-inflammatory molecules are secreted; these molecules cause or at least contribute to the accumulation of intracellular cholesterol. Therefore, it became obvious that the primary event in this sequence is not the accumulation of cholesterol but an inflammatory response.

## 1. Introduction

Formation of cells with cytoplasm filled with lipid droplets, so-called foam cells, is one of the earliest microscopically visible manifestations of atherosclerosis that occur at the level of arterial wall intima. Intracellular lipid accumulation can be regarded as a trigger event in atherogenesis at the cellular level. In turn, lipid accumulation stimulates the main atherogenic manifestations in the arterial wall, including increased cell proliferation, extracellular matrix production, and the loss of intercellular contacts [1,2,3]. According to the current understanding, circulating low-density lipoprotein (LDL) is the main source of lipids accumulating in the arterial wall cells. Noteworthy, intracellular lipid accumulation is not associated with the high levels of total cholesterol but with the atherogenic LDL cholesterol. Several authors indicated that LDL particles become atherogenic, i.e., acquire the ability to cause intracellular lipid accumulation (atherogenicity) after their chemical modification, such as desialylation, oxidation, acetylation, and others [3,4,5,6]. Meanwhile, the attempts of inducing lipid accumulation in cultured cells by incubation with native LDL were not successful. In particular, desialylation was identified as the earliest LDL modification that conveys atherogenicity and facilitates the subsequent oxidation and association of LDL particles [7,8]. Our early study demonstrated that desialylated LDL had typical characteristics attributed to other atherogenic LDL types, such as small dense and electronegative LDL [9]. To confirm these findings, further extensive studies on the atherogenic modified LDL were conducted [10,11]. These studies established that LDL particles undergo multiple atherogenic modifications in the bloodstream, including desialylation, changes of particle size and density, acquisition of negative charge, and oxidation. Moreover, modified particles were prone to the association and could induce the immune response forming large circulating immune complexes that were isolated from atherosclerotic patients’ blood and shown to be highly atherogenic [10,11]. In fact, it was found that only LDL associates, but not unassociated modified LDL particles, can be responsible for the intracellular lipid accumulation [12,13,14].

The exact mechanisms of foam cell formation remain to be fully understood, however, considering the current knowledge on the subject matter, it can be suggested that either altered LDL uptake or LDL metabolism, or both these processes may be involved. In this respect, transcriptome analysis, one of the modern experimental approaches to investigate molecular pathways of the foam cell formation, was utilized to study the genes involved in the regulation of intracellular cholesterol accumulation [15,16,17,18,19]. Thus, the top 10 master regulatory genes that may be responsible for the accumulation of cholesterol esters in cultured macrophages incubated with atherogenic modified LDL were identified in a recent study [19]. Surprisingly, it was found that most of the identified genes were involved in the regulation of inflammatory immune response but not the lipid metabolism.In contrast to the popular belief that modified LDL-induced lipid accumulation may stimulate the pro-inflammatory response, this surprising observation indicated that the pro-inflammatory response may precede the intracellular lipid accumulation. That was also observed in numerous other studies [20,21,22,23,24,25,26,27]. Accordingly, it can be hypothesized that prior to intracellular lipid accumulation, the induction of pro-inflammatory response can be triggered by the stimulation of macrophage phagocytic activity by modified LDL. This viewpoint can be also supported by the fact that native LDL cannot induce intracellular cholesterol accumulation, as is recognized by a specialized receptor on the cell surface, and therefore, it is internalized by the means of receptor-mediated endocytosis [28,29,30,31]. In this way, the lipid particle is eventually transported to the lysosome, where it can be enzymatically degraded. Whereas, modified LDL particles may not be recognized by the specialized receptors. Several studies showed that modified LDL particles form large self-associates that can be internalized by the means of non-specific cellular phagocytosis [32,33,34]. It is well known that phagocytosis is one of the early events in the innate immune response, with macrophages internalizing the pathogens in order to neutralize them. Moreover, macrophages that actively internalize foreign agents secrete pro-inflammatory cytokines facilitating the recruitment of immune cells to the area of pathogen invasion, thus, inducing the inflammatory response [35]. Therefore, it was suggested by our research team that a similar process may occur following the macrophage interaction with the large self-associates of modified LDL particles that have the size comparable to that of many pathogens [12,13,14], in the early stage of atherosclerotic plaque development (foam cell formation). Therefore, in this study, using transcriptome analysis, gene regulation in macrophages was examined to find the evidence in favor of the hypothesis that in the process of foam cell formation, upon the interaction of macrophages with modified LDL, the stimulation of phagocytosis is likely to be the primary event that, subsequently, triggering the pro-inflammatory immune response followed by the intracellular lipid accumulation.

## 2. Results

### 2.1. LDL Accumulation in Human Monocyte-Derived Primary Macrophages

Total cellular cholesterol content in monocyte-derived macrophages treated with native LDL, oxidized LDL, acetylated LDL, desialylated LDL, naturally occurring LDL, and latex beads, as well as in control (untreated) cells after 24 h incubation was presented in Table 1. All experiments were performed in triplicate. As expected, low concentrations of native LDL and latex beads did not affect the total cholesterol content in cultured macrophages. By contrast, in all samples containing modified LDL, a significant increase in total cholesterol content was observed. High-throughput sequencing of mRNA isolated from the cells treated with native, atherogenic LDL, and latex beads was performed on 21 samples from seven groups (monocytes/macrophages were obtained from three different donors in each group) (Table 1).

**Figure 1:**
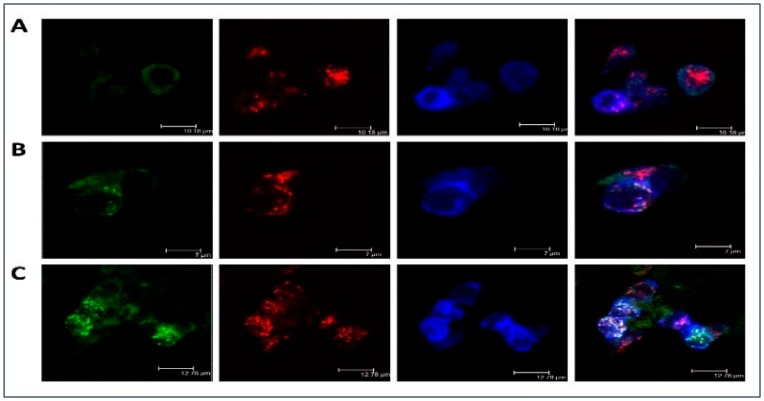
Low-density lipoprotein (LDL) accumulation in human monocyte-derived primary macrophages. Immunofluorescent staining was performed on primary human monocyte-derived macrophages and analyzed by confocal microscopy. Cells were cultured with native (**B**) or atherogenic LDL (**C**) or without LDL (**A**) for 3 h. Staining with anti-ApoB antibody was shown in green, staining with anti-Stabilin-1 was shown in red, staining with anti-EEA1 was shown in blue.

Moreover, Figure 1 illustrated LDL accumulation in cultured macrophages. As compared with the native lipoprotein, the more intense intracellular lipid accumulation was observed in the samples with atherogenic naturally occurring LDL. Besides, it was noticed that LDL was co-localized with the scavenger receptor and that may be indicative of atherogenic naturally occurring LDL associates entering the cell bypassing specialized receptors, i.e., by other means, such as phagocytosis. LDL co-localization with early endosomes and lipid accumulation caused by the atherogenic LDL was also clearly visible. That was also observed in our early study demonstrating that prolonged cell cultivation (up to 72 h) with modified LDL can lead to the occurrence of lipid inclusions in the form of dots and droplets filling the cytoplasm of cultured cells making these cells of the foamy appearance [36].

### 2.2. Search for Master Regulators

The fastq files of RNA-sequencing analysis were aligned to the reference human genome (hg19) using “HISAT2” tool with default parameters followed by the calculation of read counts done by “htseq-count” tool. As a result, we obtained a table of 19964 ENSEMBL genes with computed read counts for all samples studied here. After that, the identification of differentially expressed genes was done with the help of edgeR tool [37] applying default parameters by comparing data of each treatment with control. Table 2 showed the statistics of the identified up- and down-regulated genes (ENSEMBL IDs) applying the following cut-offs. For the up-regulated genes: logFC > 0.7 and *p*-value < 0.05; for down-regulated genes: logFC < −0.7 and *p*-value < 0.05.

**Figure 2:**
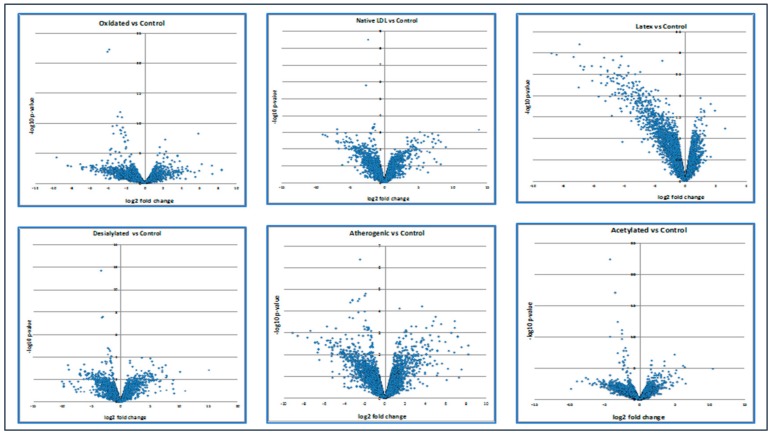
The expression level of up- and down-regulated genes compared with control. Calculations of the volcano plot can be found in the Supplementary Materials section.

Moreover, the volcano plot below demonstrated how up- and down-regulated genes were picked up (Figure 2).

**Figure 3:**
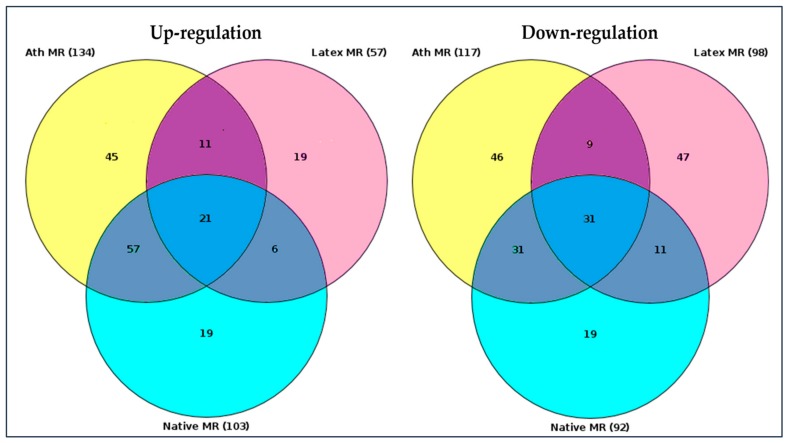
Venn diagram of master regulators identified. Native MR—master regulators identified upon the interaction (incubation) of cultured monocyte-derived macrophages with native LDL. Ath MR—master regulators identified upon the interaction (incubation) of cultured monocyte-derived macrophages with naturally occurring LDL isolated from the blood of atherosclerotic patients. Latex MR—master regulators identified upon the interaction (incubation) of monocyte-derived macrophages with latex beads. The total number of identified up- and down-regulated master regulators was shown in brackets (gene nomenclature can be found in the Supplementary Materials section). Darker colors indicated regulation of genes which coincided (diagram overlaps).

RNA sequence data were analyzed using “upstream analysis” [38,39]. Our aim was to identify the so-called master regulators in gene regulatory networks that may affect the activity of TFs that, in turn, control the differential expression of genes. The employed upstream analysis consisted of the three consequent steps, namely, (i) identification of differentially expressed genes (DEGs), (ii) analysis of promoters and TFs of DEGs, and (iii) search for potential master regulators. Only those genes, which regulation was significantly different from control samples (native LDL) were considered. Genes with a similar regulation were identified in all LDL samples, as presented in the Venn diagram, comparing the master regulators attributed to naturally occurring LDL and latex beads (Figure 3).

Out of 57 up-regulated and 98 down-regulated key genes identified for latex beads, 56 and 30 differed from the control, respectively. The genes with similar regulations were listed in Table 3. Among these genes, top 20 genes in total, 11 up-regulated and nine down-regulated master regulators were attributed to macrophage interaction as with naturally occurring LDL, as latex beads (Table 3). Out of these 20 genes, regulation of 18 genes overlapped with the regulation of genes equally changing upon macrophage interaction with LDL modified in vitro (Table 3). 

TNF receptor-associated factor 6 (TRAF6) was the only master regulator in common identified upon macrophage interaction as with naturally occurring LDL, as with all other forms of modified lipoproteins. The highest match in the number of master regulators was identified upon macrophage interaction with naturally occurring LDL and desialylated LDL (12 out of 19 master regulators). In terms of the expression of master regulators controlled by macrophage interaction with acetylated LDL, only seven master regulators were found to be in common to those attributed to macrophage interaction with naturally occurring LDL; and 11 did not match. As for master regulators controlled by macrophage interaction with oxidized LDL, 10 were the same, as compared with those attributed to macrophage interaction with naturally occurring LDL. A brief description of the known functions of matching master regulators was provided in Table 4.

Of interest, out of the top 20 master regulators identified in this study, 15 were involved in the innate immune response; 9 master regulators may be related to intracellular lipid accumulation; 10 genes were involved in the processes associated with phagocytosis (Table 5). Thus, the uppermost number of master regulators was associated with a pro-inflammatory reaction of innate immunity. These data were in good agreement with the results reported in our previous paper, where it was found that out of 10 master regulators associated with the accumulation of intracellular cholesterol, seven were associated with an inflammatory response [19].

### 2.3. The Knock-Down of Key Genes

**Figure 4:**
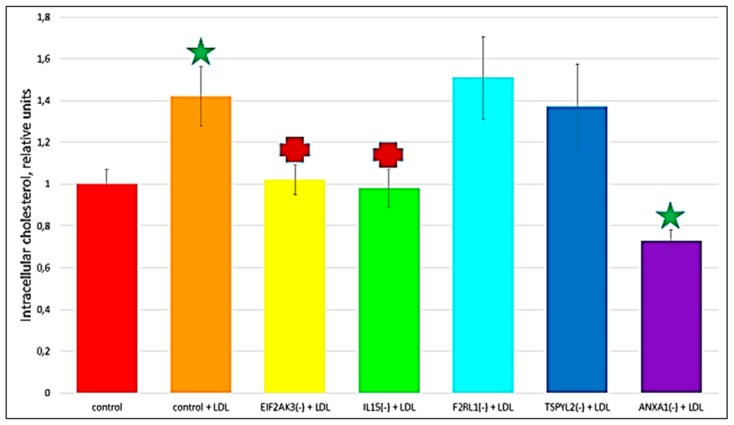
The effect of gene knock-down on cholesterol accumulation in cultured macrophages. ‘Control’, macrophages with the addition of control siRNA that did not lead to gene knock-down. + LDL, atherogenic naturally occurring LDL (50 µg protein/mL) was added to cultured cells. Star, significant difference from ‘control’ (*p* < 0.04, *t*-test and Wilcoxon–Mann–Whitney test). Cross, significant difference from ‘control + LDL’ (*p* < 0.03, *t*-test and Wilcoxon–Mann–Whitney test).

**Figure 5:**
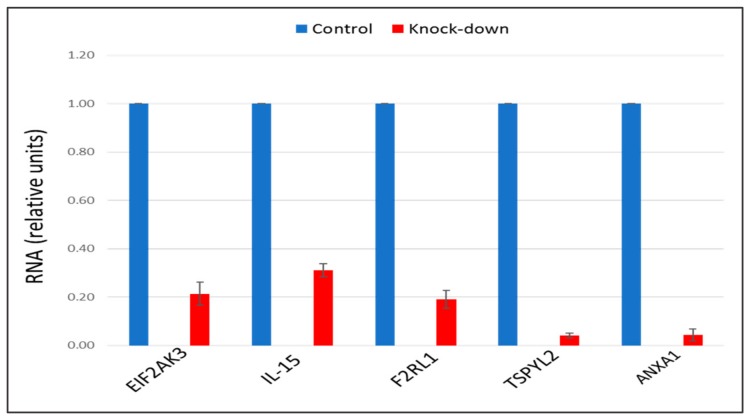
The knock-down efficiency of *EIF2AK3, IL-15, F2RL1, TSPYL2,* and *ANXA1* genes, compared with control. Genes were knocked-down by corresponding siRNA in primary macrophages. Control samples were treated with scrambled siRNA. Results presented as mean ± SD. The expression (siRNA) of *EIF2AK3, IL-15, F2RL1, TSPYL2*, and *ANXA1* genes was decreased on average by 80%, as compared to control siRNA.

The recent study conducted by our research group identified genes (*IL15, EIF2AK3, F2RL1, TSPYL2*, and *ANXA1*) encoding inflammatory molecules that were upregulated upon treatment of macrophages with atherogenic LDL and involved in the development of inflammation and immune response during the foam cell formation [19]. Further knock-down experiments using these genes were included in the present study that would help to elucidate the relationship between processes of the immune response and cholesterol accumulation. Thus, the knock-down effect of five master regulators, such as *IL15, EIF2AK3, F2RL1, TSPYL2,* and *ANXA1* on the cholesterol accumulation was tested, in order to determine, whether these genes up-regulated upon macrophage interaction with modified LDL could be involved in the accumulation of intracellular cholesterol. Primary macrophages derived from human monocytes were cultured for 24 h with the addition of atherogenic naturally occurring LDL and the significant accumulation of cholesterol was observed in these cells. The knock-down of the *EIF2AK3* and *IL15* genes completely prevented cholesterol accumulation in cultured macrophages. The knock-down of the *ANXA1* gene caused the most significant decrease in the cholesterol content (lowered basal cholesterol) in cultured macrophages. At the same time, knock-down of *F2RL1* and *TSPYL2* did not cause any effects. Figure 4 illustrated the knock-down effect of *IL15, EIF2AK3, F2RL1, TSPYL2,* and *ANXA1* genes on intracellular lipid accumulation. In addition, the knock-down efficiency of *IL15, EIF2AK3, F2RL1, TSPYL2*, and *ANXA1* genes was reflected in Figure 5.

### 2.4. Identification of the Relevant Signaling Pathways

Several signaling pathways that undergo up- and down-regulation upon macrophage interaction with LDL were identified. Since we were interested in identifying regulatory mechanisms of accumulation of intracellular lipids, the samples with native LDL, which did not produce a lipid accumulation effect, were excluded from the analysis. Thus, we only considered pathways in which regulation was significantly different from control samples (native LDL). Out of 88 up-regulated and 44 down-regulated pathways identified for latex beads, 77 and 10 differed from the control, respectively. There were identified signaling pathways attributed to both latex beads and modified LDL that regulation was changed in a similar way. Moreover, the comparison of unidirectionally-regulated pathways revealed the pathways that were similarly regulated in both using naturally occurring LDL and latex beads. Among these signaling pathways, 4 underwent up-regulation (neurotrophic signaling, *TLR2*-mediated signaling, *TLR9* pathway, *VEGF-A* pathway) and 8 were down-regulated (Aurora-B cell cycle regulation, Cdc20 deubiquitination, Cdc20 ubiquitination, cyclinB1 ubiquitination —> anaphase onset, Fzr1 ——> cyclin B1 degradation, metaphase to anaphase transition, securin degradation, Usp44 —> Cdc20). Through these signaling pathways, the master regulators may control foam cell formation.

## 3. Discussion

Herein, we have made an attempt to establish the potential mechanisms of foam cell formation in macrophage cells. Phagocytosis initiated by the atherogenic LDL appears to be an important step in foam cell formation and atherosclerosis development [3,40]. In this respect, we proposed that during foam cell formation in macrophage cells, stimulation of phagocytosis by modified LDL is likely to be the primary effect occurring upon the interaction of atherogenic modified LDL with monocytes/macrophages that, subsequently, can trigger a pro-inflammatory immune response followed by intracellular lipid accumulation. We observed the induction of phagocytosis by latex beads with a diameter of 1.1 μm, which were similar in size to LDL self-associates determined in our previous studies [12,13,14]. The latex beads were used as an unspecific phagocytosis-stimulating agent, which can be internalized independently from specialized receptors, such as immunoglobulin Fc and complement receptors. Other receptors, expressed on macrophages that can bind modified LDL particles, such as scavenger receptors (SRs), were extensively reviewed [41,42]. The macrophage scavenger receptor family has a high-affinity uptake mechanism for the lipid accumulation derived from modified LDL, therefore, it is widely accepted that they play an essential role in the foam cell formation. In recent years, the number of scavenger receptor family members raised considerably, but their redundancy makes it difficult to assess the relative contribution of each of these receptors to the process of lipid uptake. Moreover, apart from their phagocytic activity, scavenger receptors also play an important role in the innate immune recognition, as well as inflammatory signaling regulation. In this study, the highly expressed in macrophage cells scavenger receptor Stabilin-1 was used as a marker of type 2 macrophages with generally increased scavenging capacity. It was demonstrated that in macrophage cells, Stabilin-1 receptor was capable of binding and internalizing modified LDL in a functional manner. In cultured macrophages incubated with atherogenic LDL, co-localization of LDL with this receptor and subsequent lipid accumulation were clearly visible (Figure 1). That may be indicative of atherogenic naturally occurring LDL associates can enter the macrophage cell bypassing mentioned above specialized receptors, i.e., by other means, such as phagocytosis.

Furthermore, with the use of advanced bioinformatics methods, numerous master regulators (Figure 3; gene nomenclature can be found in the Supplementary Materials section) and signaling pathways (described in the Section 2.4) were identified upon macrophage interaction with LDL. It should be noted that different master regulators (up- or down-regulated) were identified for different forms of stimulating agents. Interaction of the inert phagocytosis stimulator (latex beads) and LDL forms with macrophages was expected to affect different sets of genes, relying on the fact that lipoprotein associates are complex biological supramolecular formations that can provide various stimuli to the interacting cells that cannot be duplicated by latex beads that only stimulate phagocytosis. For instance, modified LDL can induce cholesterol accumulation or interact with certain receptors. However, differing by few genes, the set of master regulators attributed to macrophage interaction with atherogenic LDL obtained from the blood of atherosclerotic patients was matching to that attributed to macrophage interaction with desialylated LDL (Table 3). This observation was not surprising because desialylation was not the only modification of LDL that occurred in vivo. In our early studies, the sequence of atherogenic LDL particle modifications was established, in which the loss of sialic acid content occurred as the earliest modification of LDL particle [7,8,10,11]. Accordingly, in this study, the process of atherogenic LDL modifications, including LDL desialylation, was induced by the incubation of native LDL with blood serum obtained from atherosclerotic patients. Continued incubation resulted in a change of the particle chemical composition, size, density, and electric charge. Consequently, modified LDL particles were prone to a spontaneous aggregation that further increased their atherogenicity, i.e., the ability to induce cholesterol accumulation in cultured cells. The LDL particles also became more susceptible to the oxidation, which appeared to be a relatively late event in the cascade of LDL particle modifications. The macrophage interaction with oxidized LDL governed the expression of master regulators, which were different from those that were controlled by the macrophage interaction with naturally occurring LDL (Table 3). This may indicate that oxidation is not a key modification occurring in the lipoprotein particle, although, it also determines its atherogenic properties. Apparently, desialylation was the primary modification. In addition, master regulators expressed upon macrophage interaction with acetylated LDL were different from that with the use of naturally occurring LDL (Table 3). Comparative analysis of master regulators involved in the interaction of modified LDL and latex beads with cultured macrophages allowed the identification of common key genes. The topmost similarity in the genetic regulation was found between the interaction of macrophages with naturally occurring LDL and desialylated LDL indicating that desialylation is the primary and most important LDL modification occurring in the human body leading to the formation of LDL self-associates that, subsequently, can trigger phagocytosis. Thus, modified LDL caused the gene regulation characteristic of the stimulation of phagocytosis. The current results can be compared to data on the effects of enzyme-modified non-oxidized LDL (ELDL) on vascular smooth muscle cells obtained by a different research group [43]. Their study demonstrated that ELDL could induce a phenotypic switch of the smooth muscle cells, accordingly, changing the expression of certain regulatory genes and inducing foam cell formation. It is, therefore, likely that modified atherogenic LDL can influence gene expression in different cell types, which can occur at different stages of atherosclerosis development. More studies are needed, to reveal common gene regulators for different cell types and different stimuli in atherosclerosis.

Interestingly, among the top 20 master regulators identified in this study, the majority (15) master regulators were found to be involved in the innate immune response (Table 5) that can be indicative of that pro-inflammatory immune response can precede lipid accumulation. That was in good agreement with several other studies that investigated the innate immune responses to modified LDL [23,25]. These studies demonstrated an increase in the expression and production of cytokines resulting from the interaction of macrophages with modified lipoproteins. Accumulating evidence suggested that it is likely that some pro-inflammatory cytokines may be involved in the formation of foam cells [44,45,46]. Moreover, here, we included further experiments testing the knockdown effect of five pro-inflammatory molecule-encoding genes identified in our previous study [19], such as *IL15, EIF2AK3, F2RL1, TSPYL2,* and *ANXA1*, on LDL accumulation, that can help to elucidate the relationship between the inflammatory immune response and the accumulation of cholesterol during the formation of foam cells (Figure 4). These genes were upregulated upon macrophage interaction with atherogenic modified LDL and were found to be associated with inflammatory immune response [19]. This study revealed that the knock-down of *EIF2AK3, IL15*, and *ANXA1* genes resulted in the complete suppression of cholesterol accumulation in cultured macrophages; and the knockdown of *ANXA1* caused the most significant decrease in the cholesterol content (lowered basal cholesterol level). Hereby, it is possible to point out that, as a result of the up-regulation of these genes, pro-inflammatory molecules can be secreted influencing the accumulation of intracellular cholesterol.

Taken together, obtained results indicated that pro-inflammatory response to phagocytosis stimulation by modified LDL precedes the accumulation of intracellular lipids and possibly contributes to the formation of foam cells. Along these lines, the sequence of events in the foam cell formation can be proposed as following: LDL particles undergo atherogenic modification that is accompanied by the formation of self-associates; large LDL associates can stimulate phagocytosis; as a result of phagocytosis stimulation, the macrophage production of pro-inflammatory molecules can occur causing or at least contributing to the accumulation of intracellular cholesterol. The molecular links between phagocytosis and the production of pro-inflammatory molecules remain obscure. In this way, the currently recognized concept of foam cell formation that accumulation of lipids triggers the pro-inflammatory response was not confirmed. Further studies are needed to explore the impact of modified LDL on the expression of secreted cytokines by the macrophages, to reveal the key signaling pathways in the local inflammatory response induced in atherosclerosis and their respective potential molecular therapeutic targets. Also, further studies are necessary for focusing on the identification of the mechanistic links between the effect of master regulator genes responding to modified LDL and atherosclerosis development. Important answers can be found by a functional genomic analysis of RNA sequencing libraries obtained from cells stimulated with modified LDL that underwent phenotypic switch or activated phagocytosis.

## 4. Materials and Methods

### 4.1. Lipoproteins and Latex Beads

Unspecific phagocytosis was induced by latex beads with mean particle size 1.1 μm (LB11, Merck, Darmstadt, Germany). Native human LDL, oxidized LDL, and acetylated LDL were obtained from Kalen Biomedical (Montgomery Village, MD, USA). Electrophoretic mobility of LDL was: 105 ± 1 pixel for native LDL, 491 ± 5 pixels for oxidized LDL, and 477 ± 5 pixels for acetylated LDL. Desialylated LDL was prepared as described previously [47]. Briefly, the samples of native LDL (normalized by total protein content 2 mg/mL) were treated with neuraminidase immobilized on agarose carrier (40 μU/mL) for 2 h at 37 °C. The treatment led to the reduction of sialic acid content in the LDL particles by approximately 70%. Then LDL samples were centrifuged for 10 min at 2500 rpm to remove agarose particles and dialyzed against phosphate-buffered saline (PBS). Naturally occurring atherogenic multiply modified LDL was isolated from atherosclerotic patients’ plasma by ultracentrifugation as described previously [19]. The patients were recruited for our study with the following mean parameters: age of 65.0 years (SD = 9.9); body mass index of 25.8 kg/m^2^ (SD = 3.9); total cholesterol of 240 mg/dL (SD = 28); LDL cholesterol of 148 mg/dL (SD = 23); HDL cholesterol of 69 mg/dL (SD = 11); triglycerides of 113 mg/dL (SD = 27); fasting glucose of 5.1 mmol/L (SD = 0.4); none of the patients had diabetes mellitus. The study was conducted in accordance with Helsinki Declaration and was approved by the institutional ethics committee.

### 4.2. Monocyte-derived Macrophages

Monocytes were isolated from peripheral blood of apparently healthy individuals using the method of plastic adhesion. All volunteers provided the written informed consent to participate in the study. Volunteers were included in the study if they had no manifestations of atherosclerosis (established coronary heart disease, acute coronary syndrome, acute myocardial infarction, peripheral atherosclerosis), hypercholesterolemia (LDL-cholesterol level exceeding 160 mg/dL or prescribed cholesterol-lowering medications), hypertriglyceridemia (triglycerides (TG) exceeding 200 mg/dL or prescribed TG-lowering medications), arterial hypertension (diastolic blood pressure >90 mm Hg and/or systolic blood pressure >140 mm Hg or prescribed antihypertensive medications), diabetes mellitus or impaired glucose tolerance (blood sugar >110 mg/dL or prescribed regular insulin injections and/or sugar-lowering medications), regular smoking (more than 10 cigarettes per day for 30 preceding months), and family history of acute myocardial infarction among first-degree relatives younger than 60 years of age. The isolated cells were incubated at 37 °C in cell culture incubator under 5% CO_2_ for 2 h and rinsed three times with RPMI-1640 culture medium to remove non-adherent cells. The adherent cells were detached mechanically and plated in culture plates (Corning, New York, NY, USA). To assess the purity of the obtained monocyte population, cells were stained with anti-CD14-conjugated antibodies and analyzed by flow cytometry. The percentage of CD14-positive cells was over 95%. Cell viability was evaluated using Trypan Blue staining and was over 98% in all experiments.

Cells were cultured in RPMI-1640 medium supplemented with 10% of human serum, 50 ng/mL human M-CSF (PeproTech, Rocky Hill, NJ, USA), and 25 ng/mL IL-10 (PeproTech, Rocky Hill, NJ, USA). Medium was refreshed on day 3 and replaced with serum-free X-VIVO medium (Lonza Group Ltd., Basel, Switzerland) on day 6. The obtained monocyte-derived macrophages were used for experiments on day 7. On day 7, X-VIVO medium was refreshed, and 50 µg/mL of LDL (normalized by total protein measured using Lowry method) or 0.4 μL/mL of latex beads suspension in X-VIVO medium were added. The addition of these agents at indicated concentrations did not influence cell viability. Cells were incubated for 24 h. After that, cells were collected for cholesterol measurement and total RNA isolation using RNeasy Plus Mini kit (Qiagen). All experiments were repeated on monocytes/macrophages isolated from 3 different donors.

### 4.3. Intracellular Cholesterol Measurement

For intracellular cholesterol measurement, cells were rinsed three times in Dulbecco’s phosphate-buffered saline containing Ca^2+^ and Mg^2+^ (DPBS). Lipids were extracted three times with hexane: isopropanol (3:2, *v/v*) mixture using the method described previously [48]. The extract was air-dried in a 96-well plate at room temperature. The obtained dry precipitate was dissolved using Amplex™ Red Cholesterol Assay Kit, according to the manufacturer’s protocol (Invitrogen, Waltham, MA, USA).

The plate was incubated at 37 °C for 30 min, and then the fluorescence was measured in a fluorescence microplate reader Synergy H4 (BioTek Instruments, Winooski, Vermont, USA) using excitation at 545 nm and emission detection at 590 nm.

After lipid extraction, cells were dissolved in 50 μL of 0.2 N NaOH and the protein content in each sample was measured using the Lowry method [49].

### 4.4. TRANSFAC and TRANSPATH Databases

Studying the transcription factor-binding sites in gene promoters was performed using a library of DNA-binding motifs obtained from the TRANSFAC^®^ database release 2019.2 (geneXplain, Wolfenbüttel, Germany) (http://genexplain.com/transfac) [50]. The identification master regulators and signaling pathways activating transcription factors were performed using a comprehensive signal transduction network based on manually curated TRANSPATH^®^ database release 2019.2 (geneXplain, Wolfenbüttel, Germany) (http://genexplain.com/transpath) [51].

### 4.5. Analysis of Enriched Transcription Factor Binding Sites in Promoters of Differentially Expressed Genes

To analyze the transcription factor-binding sites (TFBS) in the promoters of the identified differentially expressed genes, we searched for overrepresented known DNA-binding motifs using the MATCH algorithm [52]. The motifs were specified using position weight matrices (PWMs) obtained from TRANSFAC^®^ database. PWMs give weights to each nucleotide in each position of the DNA binding motif for a given transcription factor.

The frequency of TFBS identified in the promoters of differentially expressed genes was compared to that in the promoters of genes that were not expressed differentially (background sequence set). The foreground and background sequence sets were denoted briefly as “Yes” and “No” sets. We analyzed the promoter sequences of a standard length of 1100 bp (−1000 to +100 around the transcription start site (TSS)). The error rate of the site enrichment was controlled by estimating the adjusted p-value (using the Benjamini-Hochberg correction procedure) (adj. *p*-value < 0.01).

Composite modules (specific combinations of TFBS clustering inside the promoter regions) were analyzed using the Composite Module Analyst (CMA) algorithm [53] in the promoters of the “Yes” and “No” sets. We searched for composite module consisting of clusters of sites for maximum 10 transcription factors (TFs) in a sliding window of 200–300 bp that statistically significantly separated the sequences in the “Yes” and “No” sets (minimizing Wilcoxon *p*-value).

### 4.6. Identification of Master Regulators in the Signal Transduction Network

We searched for master regulator molecules in the signal transduction network upstream of the TFs identified in the analysis of promoters of differentially expressed genes. To identify the master regulators, we used a comprehensive signal transduction network of human cells (based on TRANSPATH database). The main algorithm of the master regulator search was described previously [38,54]. We searched for the nodes in the global signal transduction network that may potentially regulate the activity of the set of TFs identified at the previous step of the analysis. We computed a key-node score for each of such nodes according to the formula described in our previous publication [2]. The key-node score ranked the identified nodes according to their potential impact on the activity of the identified TFs. The nodes that could specifically regulate the activity of the maximal number of TFs from the input list were considered as the most important regulators. The algorithm was executed with a maximum radius of 12 steps upstream of each TF in the input set. The error rate of this algorithm was controlled by applying it 1000 times to randomly generated sets of input TFs of the same set-size. Z-score and FDR value of ranks were calculated for each of the nodes based on the random runs as described previously [41]. All nodes with the FDR below 0.05 were considered as potential master-regulators.

### 4.7. RNA Sequencing

Total RNA was isolated from cells and purified using NucleoSpin RNA and RNase-free DNase digestion, according to the manufacturer’s instructions (MACHEREY-NAGEL). RNA-seq libraries were prepared from poly(A)-enriched mRNA using NEBNext Ultra RNA Library Prep kit for Illumina, according to the manufacturer’s protocol (New England BioLab, Ipswich, MA, USA). Libraries were PCR-amplified for ~12 cycles and sequenced on a Hi-seq 1500 (Illumina) for 51 cycles.

### 4.8. Gene Expression Knock-Down by Small Interference RNA (siRNA)

The knock-down of genes was achieved by transfecting monocyte-derived macrophages using lipofectamine RNAiMax (Invitrogen), according to the manufacturer’s instructions. Cells were transfected with 50 nM control scrambled siRNA (Invitrogen) or target gene-specific siRNA: IL15, EIF2AK3, or F2RL1, (Santa Cruz Biotechnology, Heidelberg, Germany).

## 5. Conclusions

This study provided valuable data on the level of cellular regulatory networks in foam cell formation in support of the hypothesis that stimulation of phagocytosis by modified LDL is likely to be the primary effect occurring upon the interaction of atherogenic modified LDL with monocytes/macrophages which, subsequently, can trigger a pro-inflammatory immune response followed by intracellular lipid accumulation. The identification of possible master regulators implicated in atherosclerosis-specific regulatory networks can help in better understanding of atherosclerosis pathogenesis and finding potential specific molecular targets for its therapies.

## Figures and Tables

**Table 1:** Cholesterol content in cultured macrophages.

No	Treatment	Total Cholesterol (nmol/mg Protein)	
		Mean ± SEM	*p*-Value (Vs. Control)
1	Control	57 ± 9	-
2	Native LDL	56 ± 5	NS
3	Oxidized LDL	98 ± 11	0.014
4	Acetylated LDL	80 ± 6	0.039
5	Desialylated LDL	102 ± 8	0.004
6	Atherogenic LDL	80 ± 7	0.045
7	Latex	51 ± 11	NS

NS = Not significant.

**Table 2:** Control of cholesterol content in cultured macrophages.

No	Comparison	Number of Differentially Expressed Genes	
		Up-Regulated	Down-Regulated
2	Native LDL vs. Control	177	270
3	Oxidized LDL vs. Control	247	457
4	Acetylated LDL vs. Control	292	452
5	Desialylated LDL vs. Control	241	322
6	Atherogenic LDL vs. Control	249	366
7	Latex vs. Control	15	351

**Table 3:** Matching master regulators for LDL and latex beads.

		Qty	Gene Symbol	Gene Name
**naturally occurring LDL**	up	11	*EP300*	E1A binding protein p300
			*IQGAP1*	IQ motif containing GTPase activating protein 1
			*MAP3K14*	mitogen-activated protein kinase kinase kinase 14
			*MAP3K3*	mitogen-activated protein kinase kinase kinase 3
			*NCF2*	neutrophil cytosolic factor 2
			*PIK3R5*	phosphoinositide-3-kinase regulatory subunit 5
			*PRKCD*	protein kinase C, delta
			*PTGES*	prostaglandin E synthase
			*RAD23A*	RAD23 homolog A, nucleotide excision repair protein
			*RIPK2*	receptor interacting serine/threonine kinase 2
			*TAB1*	TGF-beta activated kinase 1/MAP3K7 binding protein 1
	dn	9	*AKT1*	v-akt murine thymoma viral oncogene homolog 1
			*CDC42*	cell division cycle 42
			*DUSP7*	dual specificity phosphatase 7
			*GSK3B*	glycogen synthase kinase 3 beta
			*HGF*	hepatocyte growth factor (hepapoietin A; scatter factor)
			*NCF1*	neutrophil cytosolic factor 1
			*TAOK1*	TAO kinase 1
			*TGFB2*	transforming growth factor beta 2
			*TRAF6*	TNF receptor associated factor 6
**desialylated LDL**	up	10	*EP300*	E1A binding protein p300
			*CASP2*	caspase 2
			*DUSP5*	dual specificity phosphatase 5
			*IQGAP1*	IQ motif containing GTPase activating protein 1
			*PIK3R5*	phosphoinositide-3-kinase regulatory subunit 5
			*PTGES*	prostaglandin E synthase
			*PRKCD*	protein kinase C, delta
			*PRKCB*	protein kinase C, beta
			*RAD23A*	RAD23 homolog A, nucleotide excision repair protein
			*RIPK2*	receptor interacting serine/threonine kinase 2
	dn	9	*DUSP7*	dual specificity phosphatase 7
			*HGF*	hepatocyte growth factor (hepapoietin A; scatter factor)
			*MAP2K5*	mitogen-activated protein kinase kinase 5
			*MAP2K7*	mitogen-activated protein kinase kinase 7
			*NCF1*	neutrophil cytosolic factor 1
			*TGFB2*	transforming growth factor beta 2
			*TGFB3*	transforming growth factor beta 3
			*TRADD*	TNFRSF1A-associated via death domain
			*TRAF6*	TNF receptor associated factor 6
**acetylated LDL**	up	4	*NCF2*	neutrophil cytosolic factor 2
			*PRKCD*	protein kinase C, delta
			*PTGES*	prostaglandin E synthase
			*SGK1*	serum/glucocorticoid regulated kinase 1
	dn	14	*AKT1*	v-akt murine thymoma viral oncogene homolog 1
			*CASP8*	caspase 8
			*DUSP1*	dual specificity phosphatase 1
			*DUSP16*	dual specificity phosphatase 16
			*GSK3B*	glycogen synthase kinase 3 beta
			*HDAC3*	histone deacetylase 3
			*IL1A*	interleukin 1 alpha
			*MAP2K1*	mitogen-activated protein kinase kinase 1
			*MAP3K1*	mitogen-activated protein kinase kinase kinase 1, E3 ubiquitin protein ligase
			*MAP4K2*	mitogen-activated protein kinase kinase kinase kinase 2
			*OTUB1*	OTU deubiquitinase, ubiquitin aldehyde binding 1
			*TAOK1*	TAO kinase 1
			*TRADD*	TNFRSF1A-associated via death domain
			*TRAF6*	TNF receptor-associated factor 6
**oxidized LDL**	up	7	*CASP2*	caspase 2
			*IQGAP1*	IQ motif containing GTPase activating protein 1
			*MAP3K14*	mitogen-activated protein kinase kinase kinase 14
			*NCF2*	neutrophil cytosolic factor 2
			*PRKCB*	protein kinase C, beta
			*RAD23A*	RAD23 homolog A, nucleotide excision repair protein
			*SGK1*	serum/glucocorticoid regulated kinase 1
	dn	13	*AKT1*	v-akt murine thymoma viral oncogene homolog 1
			*CDC42*	cell division cycle 42
			*DUSP1*	dual specificity phosphatase 1
			*DUSP16*	dual specificity phosphatase 16
			*GSK3B*	glycogen synthase kinase 3 beta
			*HDAC3*	histone deacetylase 3
			*KRAS*	Kirsten rat sarcoma viral oncogene homolog
			*MAP3K1*	mitogen-activated protein kinase kinase kinase 1, E3 ubiquitin protein ligase
			*MAP4K2*	mitogen-activated protein kinase kinase kinase kinase 2
			*MAPK8IP1*	mitogen-activated protein kinase 8 interacting protein 1
			*NCF1*	neutrophil cytosolic factor 1
			*TAOK1*	TAO kinase 1
			*TRAF6*	TNF receptor associated factor 6

Matching master regulators were listed in blue.

**Table 4:** Functions of matching master regulators identified.

Gene Symbol	Functions of Genes (According to NCBI Gene Database)
*EP300*	The adenovirus *E1A*-associated cellular *p300* transcriptional co-activator protein is encoded by this gene. It acts as histone acetyltransferase regulating transcription through chromatin remodeling that is significant in the cellular proliferation and differentiation. It mediates *cAMP*-gene regulation by specific binding to phosphorylated *cAMP* response element-binding (*CREB*) protein. It plays a role in the stimulation of hypoxia-induced genes such as *VEGF*, a co-activator of hypoxia-inducible factor 1 alpha (*HIF1A*). Mutations in this gene cause Rubinstein-Taybi syndrome and may also play a role in epithelial cancer.
*IQGAP1*	IQ Motif Containing GTPase Activating Protein 1 is a member of the *IQGAP* protein family encoded by this gene. It regulates cellular morphology and motility by interacting with components of the cytoskeleton, cell adhesion molecules, and several signaling molecules. The gene amplification-upregulated expression of this protein is attributed to two gastric cancer cell lines.
*MAP3K14*	The mitogen-activated protein kinase kinase kinase 14, a serine/threonine protein-kinase is encoded by this protein. It binds to *TRAF2* and activates *NF-kappa B*. It is involved in an *NF-kappa B*-inducing signaling cascade that is mutual to receptors of the tumour-necrosis/nerve-growth factor (*TNF/NGF*) family and to the interleukin-1 type-I receptor. It has sequence similarity with some other *MAPKK* kinases.
*MAP3K3*	It is a product of 626-amino acid polypeptide with 96.5% identity to mouse Mekk3. Its catalytic domain is interrelated to those of some other kinases, including mouse Mekk2, tobacco NPK, and yeast Ste11. A 4.6-kb transcript of this gene showing ubiquitous expression was revealed by Northern blot analysis. By activating *SEK* and *MEK1/2*, it directly regulates the stress-activated protein kinase (*SAPK*) and extracellular signal-regulated protein kinase (*ERK*) pathways, respectively. This protein does not regulate p38 pathway. As demonstrated by co-transfection assays, it can improve a transcription from a nuclear factor kappa-B (*NFKB*)-dependent reporter gene playing a role in the *SAPK* pathway.
*NCF2*	The neutrophil cytosolic factor 2, the 67-kilodalton cytosolic subunit of the multi-protein NADPH oxidase complex found in neutrophils is encoded by this gene. A burst of superoxide delivered to the lumen of the neutrophil phagosome is produced by this oxidase. Mutations in this gene, as well as in other NADPH oxidase subunits, can result in chronic granulomatous disease, a disease that causes recurrent infections by catalase-positive organisms.
*PIK3R5*	Phosphatidylinositol 3-kinases (*PI3Ks*) phosphorylate the inositol ring of phosphatidylinositol at the 3-prime position and play important roles in cell growth, proliferation, differentiation, motility, survival, and intracellular trafficking. There are three classes of PI3Ks: I, II and III, and only the class I of *PI3K*s is implicated in oncogenesis. The 101 kD regulatory subunit of the class I *PI3K* gamma complex is a dimeric enzyme that consists of a 110 kD catalytic subunit gamma and a regulatory subunit of either 55, 87 or 101 kD is encoded by this protein. By high-affinity interaction with G-beta-gamma proteins, it recruits the catalytic subunit from the cytosol to the plasma membrane.
*PRKCD*	This gene encodes a member of the protein kinase C family of serine- and threonine-specific protein kinases. Upon activation by diacylglycerol, this protein can act as both a tumor suppressor and a positive regulator of cell cycle progression. The positive or negative regulation of apoptosis is also attributed to this protein. Mutations in this gene cause the autoimmune lymphoproliferative syndrome.
*PTGES*	A glutathione-dependent prostaglandin E synthase is encoded by this gene. The expression of this gene was shown to be induced by proinflammatory cytokine interleukin 1 beta (*IL1B*). This gene expression can also be induced by tumor suppressor protein *TP53* and it may be involved in *TP53* induced apoptosis. Knockout studies in mice suggested that this gene may contribute to the pathogenesis of collagen-induced arthritis and mediate acute pain in inflammatory responses.
*RAD23A*	The protein, such as one of two human homologs of Saccharomyces cerevisiae *Rad23* is encoded by this gene. The family of *Rad23* proteins possess a modular domain structure that consists of a ubiquitin-like domain (UbL), ubiquitin-associated domain 1 (UbA1), UbA2, and XPC-binding domain. *Rad23A* plays a crucial role in nucleotide excision repair and also in the proteasome delivery of polyubiquitinated proteins.
*RIPK2*	A member of the receptor-interacting protein (*RIP*) family of serine/threonine protein kinases is encoded by this gene. The encoded protein incorporates a C-terminal caspase activation and recruitment domain (CARD). Also, it is a component of signaling complexes in both the innate and adaptive immune pathways. It is a powerful activator of NF-kappa B and can induce apoptosis in response to various stimuli.
*TAB1*	A regulator of the *MAP* kinase kinase kinase *MAP3K7/TAK1*, a mediator of various intracellular signaling pathways, including those that induced by TGF beta, interleukin 1, and WNT-1 is encoded by this gene. This protein interacts with *TAK1* kinase, thus, activating it. It was demonstrated that its C-terminal portion is sufficient for *TAK1* binding and activation, while the N-terminus portion acts as a dominant-negative inhibitor of TGF beta, suggesting that this protein may function as a regulator of TGF beta receptors and *TAK1*. It also can interact with and activate the mitogen-activated protein kinase 14 (*MAPK14/p38alpha*) representing an alternative activation pathway contributing to the biological responses of *MAPK14* to various stimuli, in addition to the *MAPKK* pathways.
*AKT1*	This gene encodes a serine-threonine protein kinase that is catalytically inactive in serum-starved primary and immortalized fibroblasts. *AKT1* along with the related *AKT2* are activated by platelet-derived growth factor. This gene is a critical mediator of growth factor-induced neuronal survival in the developing nervous system. Factors of survival can suppress apoptosis via activation of the serine/threonine kinase *AKT1*, which then phosphorylates and inactivates components of the apoptotic machinery. These gene mutations were found to be associated with Proteus syndrome.
*CDC42*	This gene encodes a small GTPase of the Rho-subfamily, which regulates signaling pathways controlling different functions of cells, including cell morphology, migration, endocytosis, and progression of the cell cycle. This protein shares some similarities with Saccharomyces cerevisiae Cdc 42 being able to complement the yeast cdc42-1 mutant. This protein can regulate actin polymerization by direct binding to Neural Wiskott–Aldrich syndrome protein (N-WASP), which, in turn, activates the actin-related protein-2/3 (Arp2/3) complex.
*DUSP7*	This gene encodes dual-specificity phosphatase 7 (*DUSP7*) belonging to a broad heterogeneous subgroup of the type I cysteine-based protein-tyrosine phosphatase superfamily that is able to dephosphorylate both tyrosine and serine/threonine residues. In particular, *DUSP7* belongs to a class of *DUSP*s, designated *MKP*s, that dephosphorylate the following: *MAPK* (mitogen-activated protein kinase) proteins *ERK* (see MIM 601795), *JNK* (see MIM 601158), and *p38* (see MIM 600289) with specificity different from that of individual *MKP* proteins. *MKP*s consist of a highly conserved C-terminal catalytic domain and an N-terminal Cdc25 (see MIM 116947)-like (CH2) domain. MAPK activation cascades mediate various cellular physiologic processes, such as proliferation, apoptosis, differentiation, and stress responses.
*GSK3B*	A serine-threonine kinase belonging to the glycogen synthase kinase subfamily is encoded by this gene. It is a negative regulator of glucose homeostasis and is involved in energy metabolism, inflammation, ER-stress, mitochondrial dysfunction, and apoptotic pathways. Defects in this gene were associated with Parkinson’s disease and Alzheimer’s disease.
*HGF*	A binding protein of hepatocyte growth factor receptor that regulates cell growth, cell motility and morphogenesis in numerous cell and tissue types are encoded by this gene. Alternative splicing results in multiple transcript variants, at least one of which encodes a preproprotein that is proteolytically processed to generate alpha and beta chains, which form the mature heterodimer. This protein is secreted by mesenchymal cells and acts as a multi-functional cytokine on cells of mainly epithelial origin. This protein also plays a role in angiogenesis, tumorigenesis, and tissue regeneration. Despite belonging to the encoded protein to the peptidase S1 family of serine proteases, it lacks peptidase activity. These gene mutations can cause non-syndromic hearing loss.
*NCF1*	This gene encodes a 47 kDa cytosolic subunit of neutrophil NADPH oxidase, which is a multicomponent enzyme that upon activation generates superoxide anion. These gene mutations can cause chronic granulomatous disease.
*TAOK1*	Thousand and one kinase 1 (*TAOK1*) protein is encoded by this gene. It is a negative regulator of *IL-17*-mediated signal transduction and inflammation. The knock-down of *TAOK1* promotes *IL-17*-induced expression of cytokines and chemokines, as well as the activation of mitogen-activated protein kinases and nuclear factor-κB. Independently of its kinase activity, it interacts with *IL-17* receptor A (*IL-17RA*), and prevents the formation of the *IL-17R-Act1* (nuclear factor activator 1, also known as tumor necrosis factor receptor-associated factor 3 interacting protein 2) complex in a dose-dependent manner.
*TGFB2*	This gene encodes a secreted ligand of the *TGF-beta* (transforming growth factor-beta) superfamily of proteins. The ligands of this family bind various TGF-beta receptors that lead to the recruitment and activation of gene expression regulating transcription factors of the *SMAD* family. Proteolytic processing of this encoded preproprotein generates a latency-associated peptide (LAP) and a mature peptide, thus, it is found in either a latent form encompassing a mature peptide homodimer, a LAP homodimer, and a latent *TGF-beta* binding protein, or in an active form encompassing exclusively the mature peptide homodimer. The mature protein may also form heterodimers with other members of the TGF-beta family. Disruption of the *TGF-beta/SMAD* pathway is associated with a variety of human cancers. A chromosomal translocation of this gene was found to be associated with Peters’ anomaly, a congenital defect of the anterior eye chamber. These gene mutations also can be associated with Loeys-Dietz syndrome.
*TRAF6*	TNF receptor-associated factor 6 (*TRAF6*) protein, a member of the TNF receptor-associated factor (TRAF) protein family is encoded by this protein. This protein consists of an amino terminal RING domain followed by four zinc-finger motifs, a central coiled-coil region, and the TRAF-C domain, a highly conserved carboxyl terminal domain. *TRAF* proteins were found to be associated with and mediate signal transduction from the TNF receptor superfamily members. *TRAF6* protein mediates signaling from the TNF receptor superfamily members, as well as members of the *Toll/IL-1* family. Signals from receptors, including CD40, *TNFSF11/RANCE*, and *IL-1* were demonstrated to be mediated by this protein. This protein is also able to interact with several protein kinases, including *IRAK1/IRAK, SRC*, and *PKC* zeta providing a link between distinct signaling pathways. *TRAF6* protein can act as a signal transducer in the NF-kappa B pathway that, in response to proinflammatory cytokines, activates I kappa B kinase (IKK). The interaction of this protein with ubiquitin-conjugating enzymes catalyzing the formation of polyubiquitin chains, such as *UBE2N/UBC13*, and *UBE2V1/UEV1A*, was found to be necessary for IKK activation. This protein can also interact with the transforming growth factor (TGF) beta receptor complex and is essential for the activation of SMAD-independent JNK and p38 kinases.

**Table 5:** Master regulators involved in the innate immune response, phagocytosis, and lipid metabolism.

Gene Symbol	Innate Immunity	Lipid Metabolism	Phagocytosis
*EP300*	+	+	-
*IQGAP1*	-	?	+
*MAP3K14*	+	?	?
*MAP3K3*	+	+	?
*NCF2*	+	-	+
*PIK3R5*	-	-	?
*PRKCD*	+	+	+
*PTGES*	+	-	-
*RAD23A*	-	-	-
*RIPK2*	+	?	+
*TAB1*	+	?	?
*AKT1*	+	+	+
*CDC42*	+	+	+
*DUSP7*	-	?	?
*GSK3B*	+	+	+
*HGF*	+	+	+
*NCF1*	+	+	+
*TAOK1*	-	?	?
*TGFB2*	+	+	?
*TRAF6*	+	?	+

The information was extracted from TRANSPATH^®^ and HumanPSD™ data banks of QIAGEN exclusively maintained, developed, and distributed by geneXplain GmbH.

## Supplementary Materials

The following are available online at https://www.mdpi.com/1422-0067/21/3/817/s1.
